# Influence of reduced N-fertilizer application on foliar chemicals and functional qualities of tea plants under *Toxoptera aurantii* infestation

**DOI:** 10.1186/s12870-022-03533-9

**Published:** 2022-04-02

**Authors:** Sabin Saurav Pokharel, Yanni Zhong, Lv Changning, Fangyuan Shen, Li Likun, Megha N. Parajulee, Wanping Fang, Fajun Chen

**Affiliations:** 1 Department of Entomology, Nanjing Agricultural University, Nanjing, 210095 China; 2 Texas A&M AgriLife Research and Extension Center, Lubbock, TX79403 USA; 3 Department of Tea Science, College of Horticulture, Nanjing Agricultural University, Nanjing, China

**Keywords:** Tea plants, Reduced-N fertilizer, Foliar chemicals and functional qualities, *Toxoptera aurantii*, Population abundance

## Abstract

**Background:**

The tea aphid, *Toxoptera aurantii* (Boyer de Fonscolombe) is a polyphagous pest predominant in tea orchards and has become the most pernicious pest deteriorating tea quality. Nitrogen (N) is essential to plant growth improvement, and it can significantly impact plant defensive ability against aphid infestation. This study was designed to quantify the influence of reduced N-fertilizer application on foliar chemicals and functional quality parameters of tea plants against the infestation of *T. aurantii*. In this study, the tea seedlings (cv. Longjing43) were applied with normal level (NL) of N-fertilizer (240 kg N ha^−1^) along with reduced N-fertilizer levels (70%NL and 50%NL), and with and without *T. aurantii* infestation.

**Results:**

The results showed that N-fertilizer application significantly affected plant biomass and photosynthetic indexes, foliar soluble nutrients and polyphenols, tea catechins, caffeine, essential amino acids, volatile organic compounds of tea seedlings, and the population dynamics of *T. aurantii*. Compared with the normal N-fertilizer level, the reduced N-fertilizer application (70%NL and 50%NL) significantly decreased all the foliar functional quality components of tea seedlings without aphid infestation, while these components were increased in tea seedlings with aphid infestation. Moreover, the transcript expression levels of foliar functional genes (including *CsTCS, CsTs1,* and *CsGT1*) were significantly higher in the NL, and significantly lower in the 50%NL for tea seedlings without aphid infestation, while the transcript expression levels were significantly higher in 50%NL in aphid inoculated tea seedlings.

**Conclusion:**

The results demonstrated that the reduced N-fertilizer application could enhance foliar chemicals and functional quality parameters of tea plants especially with *T. aurantii* infestation, which can relieve soil nitrogen pressure and reduce pesticide use for control of tea aphid infestation in tea plantations.

## Background

Tea (*Camellia sinensis* L.) is a perennial leaf-harvested crop infused from the tender leaves and buds, holds the empyrean position of the most manufactured and inexpensive beverage in the world [1–3]. As being a woody perennial plantation crop, tea is extensively cultivated as a monoculture crop in tropical and subtropical zones of Asian, African, and Latin American countries [1, 2]. There are many foliar chemicals and functional components in tea, including of polyphenols, caffeine, catechins, minerals, and some trace amounts of vitamins, amino acids and carbohydrates [3, 4], and tea also contains characteristic volatile organic compounds (VOCs) whose contribution to the overall quality of tea is of paramount importance despite comprising only 0.01% of total tea dry weight [4].

Nitrogen (N) is an indispensable element in biological systems available for plants in the form of nitrate and ammonium [5]. The management of N resource is an extremely important aspect of crop production. N is the most critical nutrient for tea plants and the elevated content is predominantly seen in young harvestable tea shoots [6]. Like most of the crops, N is responsible for the determination of quality components of tea as reported by several studies and this particular relationship is vital in the improvement of fertilizer application technology in tea production with the incorporation of integrated nutrient management which helps in maintaining or improving the tea quality [7]. Application of N fertilizer is usually practiced and is supposed to increase productivity per unit area under good cultivation practices in commercial tea plantations with recommended rates ranging from 100 kg N ha^−1^ year^−1^ to 1200 kg N ha^−1^ year^−1^ for the green tea production [8]. Ruan [9] reported that incorporation of N fertilizer might play an influential role in modifying the biochemical constituents of tea shoots while affecting the quality of specific manufactured tea products. Incorporation of N fertilizer enriches the formation and accumulation of free amino acids [10, 11] while increases or decreases the concentration of polyphenol [12] in tea shoots. Xiao et al. [13] demonstrated that N incorporation remarkably increased the caffeine concentration, while significantly decreased the concentration of soluble sugars in tea shoots [13].

Tea aphid, *Toxoptera aurantii* is a polyphagous pest predominant in tea orchards and has become the most destructive pest deteriorating tea quality [14]. *T. aurantii* normally infests succulent leaves and shoots with severe infestation leading to poor crop productivity [15]. It has also been reported that the honeydew secretion of tea aphids after sap feeding adversely impacted the photosynthetic activity of tea plants [16, 17]. According to [18], N fertilizer is a critical nutritional element which alters the severity of aphids as well as yield of the crop. And it has been reported that the elevated N fertilizer may increase the pest occurrence, explicitly of those sap feeding insects [19–21]. Vegetative growth generally increases with increased level of N fertilizer resulting in altered plant morphological characteristics which ultimately leads to pest severity[22, 23]. Augmentation of N fertilizer alone is known to be a trigger for aphid infestation with significant correlation between N fertilizer rate and level of pest infestation [24, 25]. Moreover, the lush green plants are predominantly seen after the infestation of sucking pests due to the high dose incorporation of N fertilizers [26].

The rational use of N fertilizer with sound agronomic practices that would lead to economic and ecological sustainability for tea production is a great challenge. Ameliorated N-use efficiency during crop production is vital for addressing the triple challenges of food security, environmental degradation, and climate change [27]. The exploration of using minimum N fertilizer to enhance foliar and functional quality parameters with less pest infestation and reducing environmental risks in tea plantations is the main concern for tea growers across the globe. In this study, the effects of reduced N-fertilizer application on the foliar soluble nutrients and functional components, and the emission of volatile organic compounds (VOCs) of tea plants with and without pest infestation of *T. aurantii* were studied, and the population dynamics of *T. aurantii* was investigated.

## Materials and methods

### Tea seedlings and aphid colony

The one-year-old tea seedlings (cv. Longjing43) were supplied by the Department of Tea Science, College of Horticulture, Nanjing Agricultural University, and planted in plastic pots (15 cm diameter and 13 cm depth) filled with nutrient soil (Xinggong Organic Fertilizer Co., Ltd, Zhenjiang, China). One tea seedling per pot was planted and all these potted tea seedlings were placed outside in the field condition located at the campus of Nanjing Agricultural University, Nanjing City, Jiangsu Province of China (32.01^○^N, 118.78^○^E). The tea aphids, *T. aurantii,* used in this study were also provided by the Department of Tea Science, College of Horticulture, Nanjing Agricultural University. These aphids were first reared on the one-year-old tea seedlings (cv. Longjing 43) for 3 generations in the laboratory to establish the aphid colony for the following experiments.

### N-fertilizer application

The potted experiment was conducted from Aug. 1 to Oct. 15 in 2020. Three levels of N-fertilizer application were set up, including normal (NL), 70%NL, and 50%NL. The normal level of N-fertilizer application was 0.270 g urea (i.e. N fertilizer), 0.105 g triple superphosphate (i.e. P fertilizer) and 0.105 g potassium chloride (i.e. K fertilizer) per pot according to traditionally used amounts in the local region (equivalent to 240 kg N ha^−1^, 90 kg P_2_O_5_ ha^−1^, and 120 kg K_2_O ha^−1^). The reduced N-fertilizer applications (70%NL and 50%NL) were 0.189 and 0.135 g urea with the same amount of P and K per pot, respectively. Each treatment of the N-fertilizer applications (i.e., NL, 70%NL and 50%NL) consisted of three replications, and there were 20 pots (15 cm diameter × 13 cm height) with one 1-year-old tea seedling per pot in each replication. Thus, the entire experimental setup consisted of 1 seedling/pot × 20 pots/replication × 3 replications/treatment × 3 treatments = 180 seedlings. The potted tea seedlings were watered moderately at 2-day intervals and no additional chemical fertilizers or insecticides were used during the study.

### Tea aphid inoculation

In this experiment, half of the tea seedlings (i.e., 1 seedling/pot × 10 pots/replication × 3 replications/treatment × 3 treatments = 90 seedlings) were used for the inoculation experiment of *T.* *aurantii*, while the other half (90 seedlings) were not inoculated with tea aphids and served as uninfested control group. After 45 days from the beginning of N-fertilizer application, tea aphids were inoculated on the tea seedlings with 5 T. *aurantii* adults per pot. And the population abundances and dynamics of *T.* *aurantii* (i.e., individuals per seedling) were measured for 30 days after the aphid inoculation at an interval of five days. The potted tea seedlings were watered at 2-day intervals, and no additional chemical fertilizers or insecticides were used during the entire experimental period.

### Measurement of photosynthesis of tea seedlings

After 30 days from the aphid inoculation treatment, five tea seedlings with and without *T.* *aurantii* were randomly selected from each replication of the treatments of N-fertilizer application (i.e., total 15 seedlings) respectively, which were individually measured the leaf photosynthetic parameters with a portable photosynthetic apparatus (Model: LI-6400XT; LI-COR Company, USA). The light intensity was set at 20,000 lx, and then the indexes, including net photosynthetic rate (Pn), rate of stomatal conductance (Gs), transpiration rate (Tr), and intercellular carbon dioxide (Ci) concentration of each selected tea seedlings were measured. A single mature leaf away from the terminal was randomly picked from each selected tea seedling and tested thrice and the average value was recorded.

### Measurement of the biomass of tea seedlings

After 30 days from the aphid inoculation treatment, the other five tea seedlings with and without *T.* *aurantii* were randomly selected from each replication of the treatments of N-fertilizer application (i.e., total 15 seedlings), and uprooted by the wet excavation method, and then washed with distilled water. Each individual seedling was dissected by a sharp scalpel and the portions used were root, stem and leaves. The fresh weights (root, stem and leaves) of each plant tissues of tea seedlings were measured separately using an electronic microbalance with an accuracy of ± 0.001 g (Mettler Toledo XP6, Switzerland).

### Measurement of foliar contents of soluble nutrients, functional components of tea seedlings

After measurement of the biomass, the treated tea seedlings were used to measure the foliar contents of soluble nutrients, including soluble sugars (SSs), soluble proteins (SPs), and free fatty acids (FFAs) and functional components, including amino acids (AAs), total polyphenols (TPs), volatile organic compounds (VOCs), and tea catechins.

#### Foliar soluble nutrient contents

The foliar soluble components (SSs, SPs and FFAs) of tea seedlings inoculated with and without *T.* *aurantii* were quantified in this study. For the determination of FFAs and SSs, one gram (g) of leaf fresh samples were cut and put into 5 ml of 80% ethanol and the mixture was kept in hot water bath for 10 min at 100 ℃, and then centrifuged at 13,000 rpm for 10 min. The supernatant thus formed was collected, and the pellet was re-extracted in 5 ml of 80% ethanol at 70℃, and then the pellet was re-extracted in 5 ml of 80% ethanol at 70 ℃, finally the supernatant was collected [28]. The supernatants were pooled and the foliar SS contents were measured by an enzymatic analysis using phenol sulfuric acid [29, 30]. The foliar FFAs content was determined in the remaining supernatant according to the method devised by [31] using leucine as the standard [32]. While the foliar SP content was measured according to the [33], in which 5 ml of the protein reagent was added into 0.1 ml of the extraction and the contents were mixed on the vortex mixture. The relative absorbance was measured at 595 nm (nm) after 1 h. The foliar SP content was calculated from a constructed standard curve for bovine serum albumin. The estimation of the foliar FFAs content was carried out following the extraction procedure of [34], and transformed into methyl esters using the method devised by [35], and then quantified using gas chromatography [36] equipped with an flame ionization detector (FID) detector [37, 38].

#### Foliar functional contents

##### Amino acids(AAs)

The foliar contents of AAs, including theanine (The), aspartic acid (Asp), arginine (Arg), Threonine (Thr), serine (Ser), alanine (Ala), glutamic acid (Glu), glycine (Gly), isoleucine (Ile), histidine(His), lysine (Lys) of tea seedlings inoculated with and without T. aurantii were measured with the addition of 5 ml of tea leaf extract with 5 ml of sulfosalicylic acid and centrifuged the mixture at 13,000 rpm for 5 min to facilitate the chemical reaction. The mixture was then filtered through a 0.20 µm nylon filter membrane and run using an automatic amino acids analyzer (Hitachi L-8900, Japan).

##### Total Polyphenols (TPs)

Folin-Ciocalteu colorimetric method [39] was used in the determination of tea leaf extract’s phenolic content. The sample extracts were diluted 1:20 with distilled water to obtain readings within the standard curve ranges of 0.0–600.0, lg of gallic acid per ml. The tea leaf extracts were oxidized with the Folin- Ciocalteu reagent, and the reaction was neutralized with sodium carbonate for 90 min at room temperature; the relative absorbance was measured at 760 nm by an MRX II Dynex plate reader (Dynex Technologies Inc.VA, USA). The absorbance values were then compared with those of standards with known gallic acid concentrations. All values were stated as the mean (mg gallic acid equivalents per g tea leaf extract) ± SD for three replications.

##### Tea catechins

The foliar contents of catechins, including gallocatechin gallate (GC), epigallocatechin (EGC), epigallocatechin gallate (EGCG), epicatechin (EC) and catechin gallate (CG), were quantified using the procedure described by [40]. The collected leaf samples were dried at 80 ℃ for 24 h, and the catechins thus present in the tea were extracted with water: ethanol (3:7) by sonication for 20 min (min). Then the high-performance liquid chromatography (HPLC) was performed after the centrifugation of extraction solution. The column thus used was a CAPCELL PAK C18 MG (4.6 mm I.D. x 150 mm) and gradient elution at constant column temperature with 30 °C under UV detector with 210 nm. Mobile phase A was 0.1% H3PO4 in water, and mobile phase B was 0.1% H3PO4 in methanol with the flow rate 1 ml/min. The quantification of catechins was performed by quantifying the areas of standardization and the results thus obtained in µgL–1 were expressed in % for catechins.

##### Caffeine

The tea leaf samples were dried at 80 °C for 24 h, and then caffeine was extracted and purified from dried tea leaves. The foliar caffeine content was quantified using an HPLC-based method [41].

##### Volatile organic compounds (VOCs)

The VOCs in leaves were collected using the headspace solid-phase micro extraction (HS-SPME) method combined with gas chromatography mass spectrometry (GC–MS) from the tea seedlings inoculated with and without T. aurantii for 30 days and grown under the normal and reduced N-fertilizer levels for 45 days, respectively. Approximately 2.0 g fresh weight of tea leaves were randomly selected from each treatment, weighed and placed in a 40 ml brown glass vial capped with a polytetrafluoroethylene septum and an aluminum cap (Agilent Technologies, Inc., California, PA, USA). An SPME fiber coated with 100 μm polydimethylsiloxane (PDMS, Supelco Inc., Bellefonte, PA, USA) was used for extraction under the equilibrium time of 15 min at 60 °C [42]. The analysis was performed using a gas chromatography-mass spectrometry (GC–MS, 7890A-5975C, Agilent Technologies, Inc., California, PA, USA) and equipped with an HP-5 MS capillary column (30 m × 0.25 mm i.d. × 0.25 μm film). The oven temperature program was as follows: initial temperature at 50 °C for 2 min, and then programmed at 4 °C min−1 to 140 °C held for 10 min, then at 5 °C min−1 to the final temperature of 220 °C, which was held for 5 min. The injection temperature and ion source temperature were 250 °C and 230 °C, respectively. Helium (99.999%) was the carrier gas at a flow-rate of 1 mL min−1. The ionizing energy was 70 eV, and the mass range scanned were 40–500 amu in the full-scan acquisition mode. Then the compounds were tentatively identified on the basis of their retention times and by interpretation of MS fragmentation patterns with those of standard libraries NIST08, and those reported in the specialized literature [42, 43]. Finally, the amounts of the detected volatiles were tabulated and analyzed based on comparison of their peak areas.

### Bioassay of the transcript expression levels of synthetic genes of foliar functional components

After measurement of the biomass, and the foliar contents of soluble nutrients and functional components, the treated tea seedlings were used to measure the transcript relative expression levels of four key synthetic genes of the foliar functional components, including caffeine synthase gene (*CsTCs*), theanine synthase gene (*CsTs1*) and VOCs related gene for aroma; glycosyltransferase1 (*CsGT1*).

#### RNA preparation and reverse transcription

The total RNA was extracted from the leaf samples of tea seedlings inoculated with and without *T.* *aurantii* and grown under the normal and reduced N-fertilizer levels for 45 days, by using the Trizol reagent (Invitrogen). The concentration and quality of the samples were determined by NanoDrop™ Spectrophotometer (Thermoscientific) and 1.5% agarose gel electrophoresis. The 1st strand complementary Cdna templates were synthesized with 100 ng total RNA by using the PrimeScript™ RT reagent kit with Gdna Eraser (TaKaRa, Japan). Reverse transcriptase reactions were performed in a 20 µl final volume reaction.

#### Real-time PCR analysis

Each cDNA product from the leaf samples of tea seedlings inoculated with and without *T.* *aurantii* for 30 days and grown under the normal and reduced N-fertilizer levels for 45 days was diluted twice from 20% to 1.35% solution using RNase –free dH_2_O, in order to make the Ct value within the feasible range of 15–35 based on preliminary experiments. For the fluorescence based quantitative real –time PCR (qrt-PCR), 2 µl Cdna dilution and 0.2 µM primer were used in 1*SYBR premix Ex Taq tm (TaKaRa, Japan) with the 7500 Real-Time PCR detection System (Applied Biosystems, Foster City, CA) following the supplier’s instructions. The reactions were performed in a 20 µl final volume. Then, the specific primers were designated using NCBI, and the reference gene *CsGADPH* [44] was used as the internal standard to analyze the transcript expression levels of five genes of foliar functional components in tea seedlings inoculated with and without *T.* *aurantii* for 30 days, after grown under the normal and reduced N-fertilizer levels for 45 days, respectively. The selected foliar functional genes were caffeine synthase gene (*CsTCs*) [44], theanine synthase gene (*CsTs1*) [45] and glycosyltransferase 1 (*CsGT1*) [46]. All primers used for the qRT-PCR analysis were shown in Table 1. Quantification of the relative transcript expression levels of measured genes was conducted following the 2 ^–ΔΔCt^ normalization method described by [47]. The relative transcript expression levels of reference gene (i.e., internal control) gene *CsGDPAH* were examined in every PCR plate to eliminate systematic errors. For each biological replicate (nine replicates per nitrogen treatment), three technical repeats were performed in the qRT-PCR analysis.

**Table 1:** Primers used for the qRT-PCR analysis of the gene transcript expression levels of foliar functional components in tea seedlings

S/No	Primer Name	Primer	References
1	*CsGDPAH-q-F*	TTGGCATCGTTGAGGGTCT	[48]
	*CsGDPAH-q-R*	CAGTGGGAACACGGAAAGC	
2	*CsTCS-q-F*	AGTGGCCTCAATGGCACAGC	[44]
	*CsTCS-q-R*	TGTTTGGACCCGCTGCACAA	
3	*CsTs1-q-F*	TCTGAGTCTGCCCGAACCCA	[45]
	*CsTs1-q-R*	ATGCAATCTCCGCCTTGCGA	
4	*CsGT1-q-F*	CGTCCGATCGTGATGCCACT	[46]
	*CsGT1-q-R*	CCCAGCTTCTTCTGCGGCTT

**Table 2:** Two-way ANOVAs for the effects of the interaction between N-fertilizer application and aphid infestation on biomass per plant and root: shoot ratio (RSR), photosynthetic indexes, foliar contents of soluble nutrients, functional components, and the gene transcript expression levels of tea seedlings inoculated with and without *Toxoptera aurantii *for 30 days, after 45 days grown under normal and reduced N-fertilizer application levels (*F*/*P* values)

Measured indexes			fertilizer application (N)	Aphid infestation (Aphid)	N×Aphid
Dry biomass per plant (g)	Root		32.01/<0.001^***^	188.48/<0.001^***^	72.11/<0.001^***^
	Stem		15.25 /<0.001^***^	87.61/<0.001^***^	34.56/<0.001^***^
	Leaves		22.41/<0.001^***^	292.76/<0.001^***^	41.02/<0.001^***^
	Shoot (stem and leaves)		22.52 /<0.001^***^	178.69/<0.001^***^	45.37/<0.001^***^
	Total		20.70 /<0.001^***^	120.83/<0.001^***^	39.64/<0.001^***^
Root shoot ratio (RSR)			0.75/0.493	16.49/<0.001^***^	0.02/0.983
Photosynthetic indexes	Pn (µmol CO_2_ m^-2^s^-1^)		1.37/0.288	0.00/0.993	6.15/0.013^*^
	Gs (molH_2_O m^-2^ s^-1^)		2.54/0.120	7.97/0.015^**^	56.78/<0.001^***^
	Ci (µmol CO_2_ mol ^-1^)		0.7/0.511	0.25/0.62	2.13/0.159
	Tr (mol H_2_O m^-2^ s^-1^)		0.76/0.484	0.05/0.84	12.26/<0.001^***^
Foliar soluble nutrients	Soluble Sugars (SSs; µg/g)		2.3/0.142	8.38/0.013^**^	46.71/<0.001^***^
	Soluble proteins (SPs; µg/g)		12.91 /<0.001^***^	62.62/<0.001^***^	53.43/<0.001^***^
	Free Fatty Acids (FFAs; µg/g)		11.46 /<0.001^***^	22.3/<0.001^***^	86.63/<0.001^***^
Foliar functional components	Total polyphenols (TPs; mg/g)		20.70/<0.001^***^	120.83/<0.001^***^	39.64/<0.001^***^
	Theanine (Th)		562.04/<0.001^***^	0.82/0.384	1825/<0.001^***^
	Catechins (%)	Gallocatechin gallate (GC)	6.18/0.014^*^	70.67/<0.001^***^	115.05/<0.001^***^
		Epigallocatechin (EGC)	0.008/0.992	23.82/<0.001^***^	133.97/<0.001^***^
		Epigallocatechin gallate (EGCG)	0.08 /0.926	18.79/<0.001^***^	138.39/<0.001^***^
		Epicatechin (EC)	2.09/0.166	1.88/0.196	48.93/<0.001^***^
		Catechin gallate (CG)	0.25/0.783	1.73/0.213	44.69/<0.001^***^
	Caffeine (mg/g)		0.82/0.466	0.00/1	297.96/<0.001^***^
Free Amino Acids (FAAs; ng/20µl)	Aspartic acid (Asp)		1.98/0.181	1.14/ 0.307	28.00/<0.001^***^
	Threonine (Thr)		2.22/0.151	4.94/0.046	33.78/<0.001^***^
	Serine (Ser)		1.86/0.199	1.14/0.307	40.89/<0.001^***^
	Glutamic acid (Glu)		0.56/0.584	1.14/0.307	793.04/<0.001^***^
	Glycine (Gly)		4.8/0.029	26.24/<0.001^***^	40.89/<0.001^***^
	Ala		84.87<0.001^***^	125.94/<0.001^***^	203.18/<0.001^***^
	Isoleucine (Isl)		1.62/ 0.239	2.44/0.144	51.08/<0.001^***^
	Lysine (Lys)		29.08/<0.001^***^	623.16/<0.001^***^	200.73/<0.001^***^
	Histidine (His)		654.89/<0.001^***^	3.09/0.104	2313/<0.001^***^
	Arginine (Arg)		472.62<0.001^***^	1.70/0.216	931.19/<0.001^***^
Transcript expression levels of genes	*CsTCs*		8.44/0.005	259.708/<0.001***	68.71/<0.001***
	*CsTs1*		7.82/0.006	192.21/<0.001***	59.49/<0.001***
	*CsGT1*		1.14/0.35	95.41/<0.001***	50.46/<0.001***
Measured VOCs	Toluene (TOL)		35.75/<0.001^***^	99.94/<0.001^***^	139.48/<0.001^***^
	Ethylbenzene (ETB)		0.90/0.433	59.62/<0.001^***^	33.70/<0.001^***^
	O-xylene (OXL)		0.84/0.457	31.10/<0.001^***^	58.68/<0.001^***^
	2,2, 7,7- tetramethyloctane (TMO)		97.96/<0.001^***^	2435/<0.001^***^	664.05/<0.001^***^
	Decane (DEC)		15.57/<0.001^***^	42.26/<0.001^***^	81.02/<0.001^***^
	Dodecane (DODEC)		5.49/0.020	26.47/<0.001^***^	61.94/<0.001^***^
	Azulene (AZU)		29.11/<0.001^***^	126.76/<0.001^***^	145.05/<0.001^***^
	Di-epi-alphacedrene (DEACD)		4819/<0.001^***^	18730/<0.001^***^	5698/<0.001^***^
	2,4-di-tert-butylphenol (DTP)		12.30/<0.001^***^	18.62/<0.001^***^	134.92/<0.001^***^
	1,3 benzenedicarboxylic acid (BDCXA)		1759/<0.001^***^	2186/<0.001^***^	2850/<0.001^***^

N-fertilizer application - The normal N-fertilizer application level (NL), and two reduced N-fertilizer application levels of 70% and 50% NL, Aphid inoculation - Aphid infestation and non-infestation; ^*^ *P*<0.05, ^**^ *P*<0.01, and ^***^ *P*<0.001

### Data analysis

IBM-SPSS v 20.0 (IBM, Armonk, NY USA) was used for analyzing all the experimental data. The data of the measured indexes of biomass (aboveground and belowground) and RSR, photosynthetic indexes (including Pn, Gs, Ci, Tr), foliar soluble nutrients (including SSs, SPs, FFAs) and tea functional components (including AAs, TPs, catechins (GC, EGC, EGCG, EC, CG), caffeine, and VOCs), and the relative transcript expression levels of the measured four genes (*CsTCs, CsTs1* and *CsGT*_*1*_*)* were analyzed by using two-way analysis of variance (ANOVA) with N-fertilizer application levels (NL, 70%NL and 50%NL) and aphid inoculation (inoculated and non-inoculated control) as sources of variability, and significant differences in the measured indices between/among treatments were analyzed by the LSD test at *P* < 0.05. Moreover, two-way repeated-measures ANOVA was also used with sampling time as repeated measures to analyze the effects of N-fertilizer application and aphid inoculation on the population dynamics of *T.* *aurantii* and significant differences between/among treatments was analyzed by the paired-t test at *P* < 0.05. Additionally, the Pearson’s test was performed by using R software (version R i386 3.42) to analyze the correlation between the abundances of *T.* *aurantii* and the biomass, foliar nutrients/functional components and relative transcript levels of four genes (*CsTCs, CsTs1* and *CsGT*_*1*_) of tea seedlings.

## Results

### Plant biomass of tea seedlings with and without aphid infestation under normal and reduced N-fertilizer applications

**Fig. 1:**
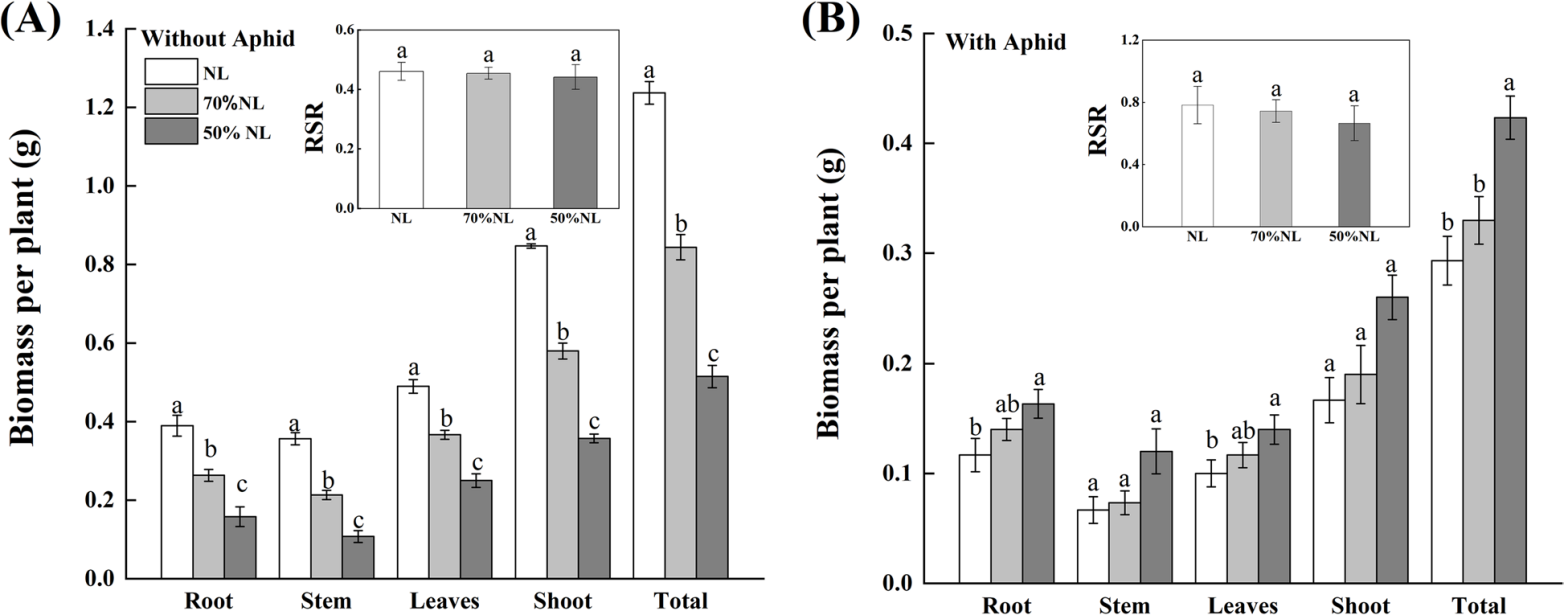
Plant biomass and root-shoot ratio (RSR) of tea seedlings without (**A**) and with (**B**) *Toxoptera aurantii* infestation for 30 days after 45 days grown under normal and reduced N-fertilizer levels (Note: RSR—root: shoot ratio; NL, 70%NL and 50%NL—100%, 70% and 50% of normal N-fertilizer level, respectively; Different lowercase and uppercase letters indicated significant difference among NL, 70%NL and 50%NL for tea seedling with or without aphid infestation, and between aphid infestation and no aphid infestation for tea seedling grown under same N-fertilizer level by the LSD test at *P* < 0.05. The same denotations will be followed latter)

N-fertilizer application, aphid infestation and their interaction all significantly affected the biomass of root, stem, leaves, shoot (including stem and leaves) and total tea seedling except root to shoot ratio (i.e., RSR) of tea seedlings (*P* < 0.001; Table 2). Compared with normal N-fertilizer level (i.e., NL), reduced N-fertilizer levels (i.e., 70%NL and 50%NL) significantly reduced the biomass of root stem, leaves, shoot and total tea seedling without aphid infestation (*P* < 0.05), and the biomass of plant tissues of tea seedlings in 50%NL was significantly lighter than that in 70%NL when there was no aphid infestation (*P* < 0.05; Fig. 1A) while 50%NL significantly increased the biomass of root, leaves and total tea seedling with aphid infestation (*P* < 0.05; Fig. 1B).

### Photosynthetic indexes of tea seedlings with and without aphid infestation under normal and reduced N-fertilizer applications

**Fig. 2:**
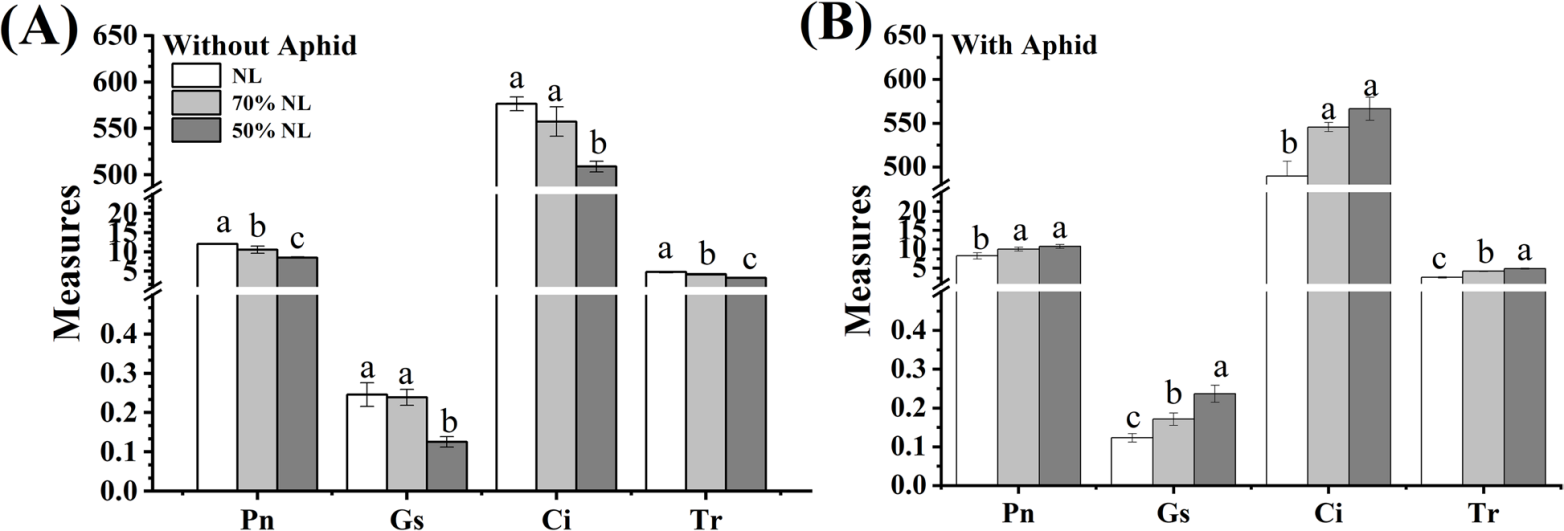
Photosynthetic indexes: net photosynthetic rate (Pn; µmol CO_2_^m−2^ s^−1^), stomatal conductance (Gs; mol H_2_O m^−2^ s^−1^), intercellular CO_2_ concentration (Ci; µmol CO_2_ mol ^−1)^ and transpiration rate (Tr; mol H_2_O m^−2^ s^−1^) in the leaves of tea seedlings without (**A**) and with (**B**) *T*. *aurantii* infestation for 30 days after 45 days grown under normal and reduced N-fertilizer levels

Aphid infestation significantly affected the stomatal conductance (Gs) in the leaves of tea seedlings (*P* = 0.015 < 0.05), and there were significant interactions between N-fertilizer application and aphid infestation on the photosynthetic indexes of net photosynthetic rate (Pn; *P* = 0.013 < 0.05), transpiration rate (Tr) and Gs (*P* < 0.001; Table 2). And the values of all the measured photosynthetic indexes were reduced with the decrease of N-fertilizer level for tea seedlings without aphid infestation (Fig. 2A); the response was opposite for tea seedlings with aphid infestation (Fig. 2B).

Compared with normal (NL), reduced fertilizer levels (70%NL and 50%NL) both significantly reduced the values of Pn (-12.22% and -29.29%) and Tr (-11.82% and -29.11%), and 50%NL also significantly decreased the values of Gs (-48.99%) and Ci (-11.73%) in the leaves of tea seedlings without aphid infestation (*P* < 0.05; Fig. 2A); while 70%NL and 50%NL both significantly increased the values of Pn (+ 17.78% and + 23.09%), Gs (+ 28.09% and + 48.02%), Ci (+ 10.26% and + 13.56%) and Tr (+ 36.13% and + 44.72%) in the leaves of tea seedlings with aphid infestation (*P* < 0.05; Fig. 2B). Moreover, the values of Pn, Gs, Ci and Tr in the leaves of tea seedlings without aphid infestation under 50%NL were significantly lower than those under 70%NL (*P* < 0.05; Fig. 2A), while the values of Gs and Tr in the leaves of tea seedlings with aphid infestation under 50%NL were significantly higher than those under 70%NL (*P* < 0.05; Fig. 2B).

### Foliar soluble nutrients and functional components of tea seedlings with and without aphid infestation under normal and reduced N-fertilizer applications

#### Foliar soluble nutrients and total polyphenols

N-fertilizer application, aphid infestation and their interaction all significantly affected the foliar contents of soluble proteins (SPs) and free fatty acids (FFAs) of tea seedlings (*P* < 0.001), and aphid infestation (*P* = 0.013 < 0.05) and its interaction with N-fertilizer application (*P* < 0.001) significantly affected the foliar contents of soluble sugars (SSs) of tea seedlings (Table 2).

**Fig. 3:**
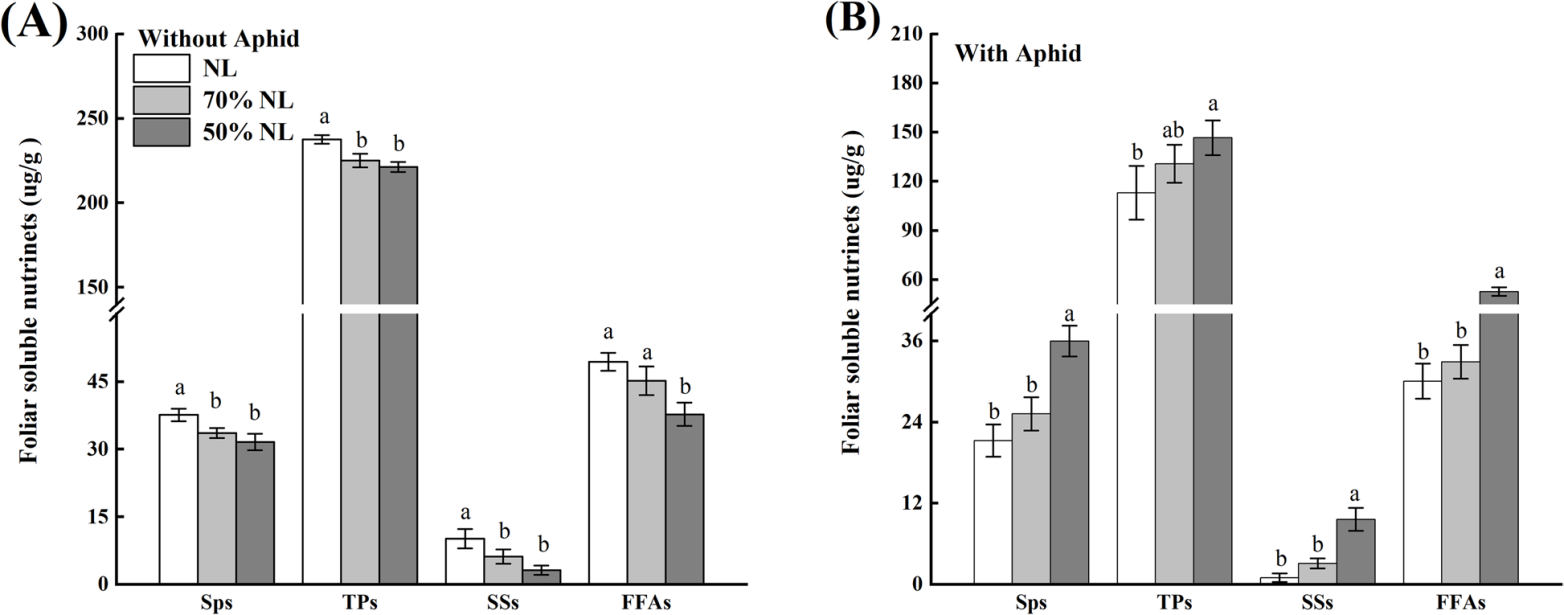
Foliar contents of soluble nutrients including soluble proteins (SPs; µg/g), soluble sugars (SSs; mg/g), free fatty acids (FFAs; µg/g)), and total polyphenols (TPs; mg/g) of tea seedlings without (**A**) and with (**B**) *T*. *aurantii* infestation for 30 days after 45 days grown under normal and reduced N-fertilizer levels

Compared with NL, 50%NL significantly decreased the foliar contents of SPs (-15.90%), SSs (-68.95%) and FFAs (-23.58%) of tea seedlings without aphid infestation, and 70%NL also significantly reduced the foliar content of SSs (-39.24%) of tea seedlings without aphid infestation (*P* < 0.05; Fig. 3A), whereas 50%NL significantly increased the foliar contents of SPs (+ 40.89%), SSs (+ 89.81%) and FAAs with aphid infestation (+ 42.96%; *P* < 0.05, Fig. 3B).

#### Foliar functional components

##### Total polyphenols

N-fertilizer application, aphid infestation and their interaction all significantly affected the foliar contents of total polyphenols of tea seedlings (TPs; P < 0.001, Table 2), Compared with NL, 70%NL and 50%NL both reduced the foliar TPs content of tea seedlings without T. aurantii infestation (-5.28% and -6.88%; P < 0.05, Fig. 3A), while 50%NL and 70% NL significantly enhanced the foliar TPs content of tea seedlings with T. aurantii infestation (+ 22.88%, + 13.51%; P < 0.05, Fig. 3B) respectively.

##### Theanine

N-fertilizer application and its interaction with aphid infestation both significantly affected the foliar theanine content of tea seedlings (P < 0.001, Table 2). Compared with NL, reduced levels of nitrogen (70%NL and 50%NL both) significantly decreased the foliar theanine contents of tea seedlings without T. aurantii infestation (-3.86% and -59.81%; P < 0.05, Fig. 6A), whereas significantly increased the foliar theanine contents of tea seedlings with T. aurantii infestation (+ 58.58% and + 57.92%; P < 0.05, Fig. 6B). Moreover, the foliar theanine content of tea seedlings for the 50%NL was significantly lower than that for the 70%NL under no aphid infestation (P < 0.05; Fig. 6A). The foliar theanine contents of tea seedlings with aphid infestation were increased by 58.58% in 70%NL and 57.92% in 50%NL, as compared to that in NL.

##### Tea catechins

N-fertilizer application significantly affected the foliar content of gallocatechin gallate (GC) of tea seedlings (TPs; P = 0.014 < 0.05), whereas aphid infestation significantly affected the foliar contents of GC, epigallocatechin (EGC) and epigallocatechin gallate (EGCG) of tea seedlings (P < 0.001); the interaction between N-fertilizer application and aphid infestation significantly influenced the foliar contents of catechin gallate (CG), epicatechin (EC), GC, EGC and EGCG of tea seedlings (P < 0.001; Table 2).

**Fig. 4:**
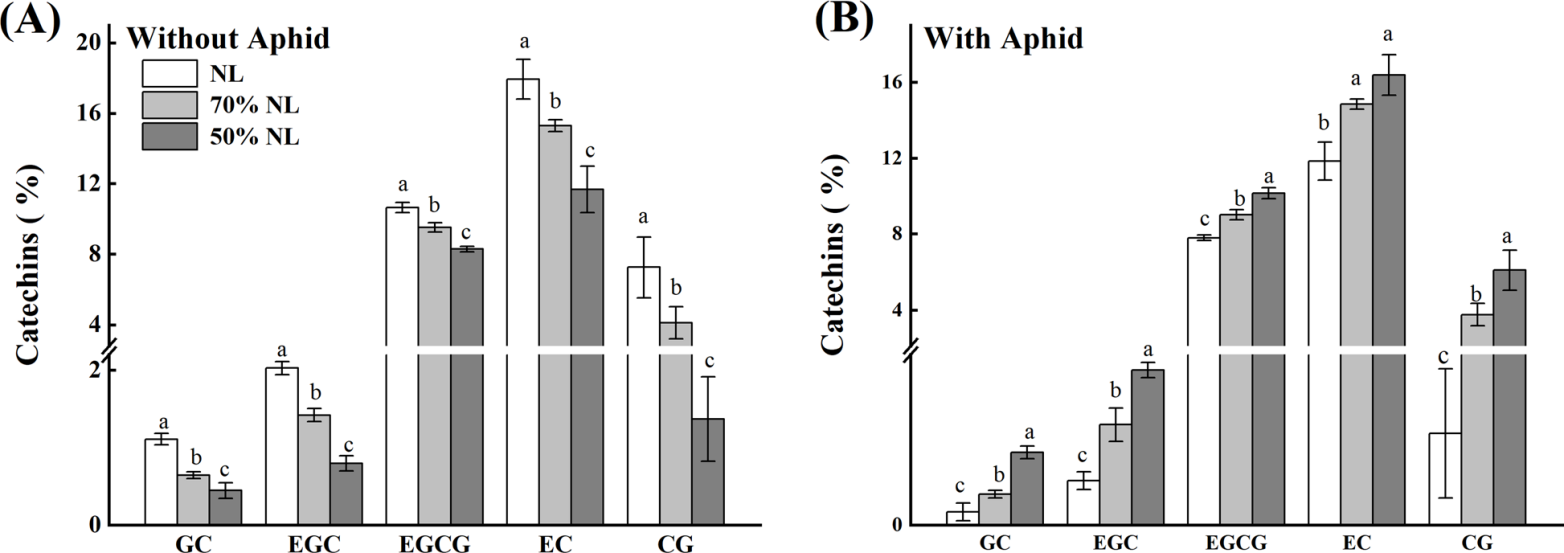
Percentage concentration of foliar functional components of tea catechins (including gallocatechin gallate (GC), epigallocatechin (EGC), epigallocatechin gallate (EGCG), epicatechin (EC), catechin gallate (CG)) of tea seedlings without (**A**) and with (**B**) *T*. *aurantii* infestation for 30 days after 45 days grown under normal and reduced N-fertilizer levels

Compared with NL, 70%NL and 50%NL both significantly decreased the foliar contents of CG, EC, GC, EGC and EGCG of tea seedlings without T. aurantii infestation (P < 0.05; Fig. 4A), whereas the reduced N treatments significantly increased the foliar contents of CG, EC, GC, EGC and EGCG of tea seedlings with T. aurantii infestation (P < 0.05; Fig. 4B). Moreover, the foliar contents of CG, EC, GC, EGC and EGCG of tea seedlings for the 50%NL were significantly lower than those for the 70%NL under no aphid infestation (P < 0.05; Fig. 4A), and significantly higher than those except that of EC for the 70%NL under aphid infestation (P < 0.05; Fig. 4B).

##### Caffeine

**Fig. 5:**
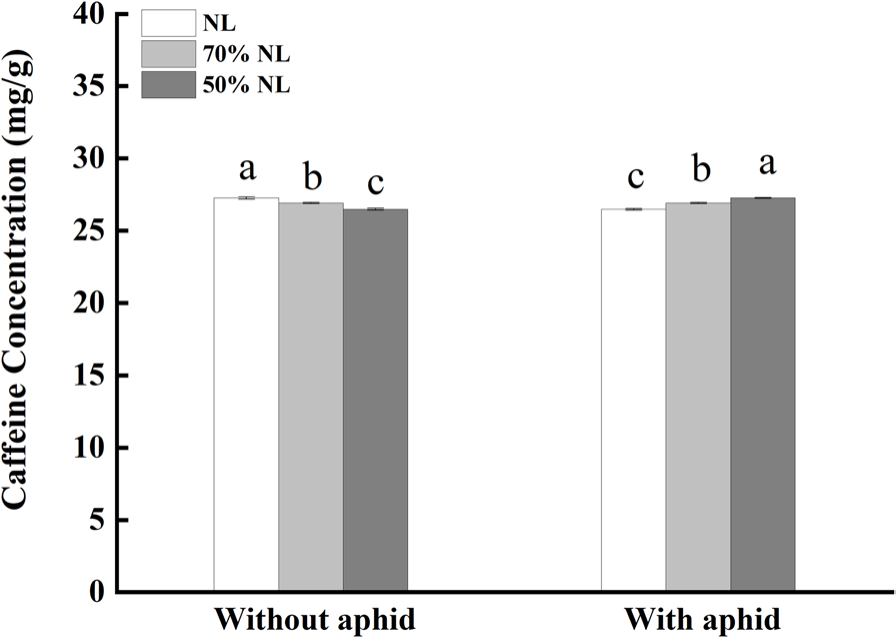
Foliar contents of the functional component, caffeine (mg/g) of tea seedlings without and with *T*. *aurantii* infestation for 30 days after 45 days grown under normal and reduced N-fertilizer levels

There was a significant interaction between N-fertilizer application and aphid infestation on the foliar caffeine content of tea seedlings (P < 0.001; Table 2). Compared with NL, 70%NL and 50%NL both significantly reduced the foliar caffeine contents of tea seedlings without T. aurantii infestation (-1.30% and -2.86%; P < 0.05, Fig. 5), but the reduced N levels significantly increased the foliar caffeine contents of tea seedlings with T. aurantii infestation (+ 1.61% and + 2.86%; P < 0.05, Fig. 5). Moreover, the foliar caffeine content of tea seedlings for the 50%NL was significantly lower than that for the 70%NL under no aphid infestation (P < 0.05; Fig. 5), and significantly higher than that for the 70%NL under aphid infestation (P < 0.05; Fig. 5).

#### Essential amino acids (AAs)

N-fertilizer application significantly affected the foliar contents of alanine (Ala), lysine (Lys), histidine (His) and arginine (Arg) of tea seedlings (*P* < 0.001), aphid infestation significantly influenced the foliar contents of glycine (Gly), Ala and Lys of tea seedlings (*P* < 0.001), and the interaction between N-fertilizer application and aphid infestation significantly affected the foliar contents of all the essential amino acids (AAs; *P* < 0.001, Table 2).

**Fig. 6:**
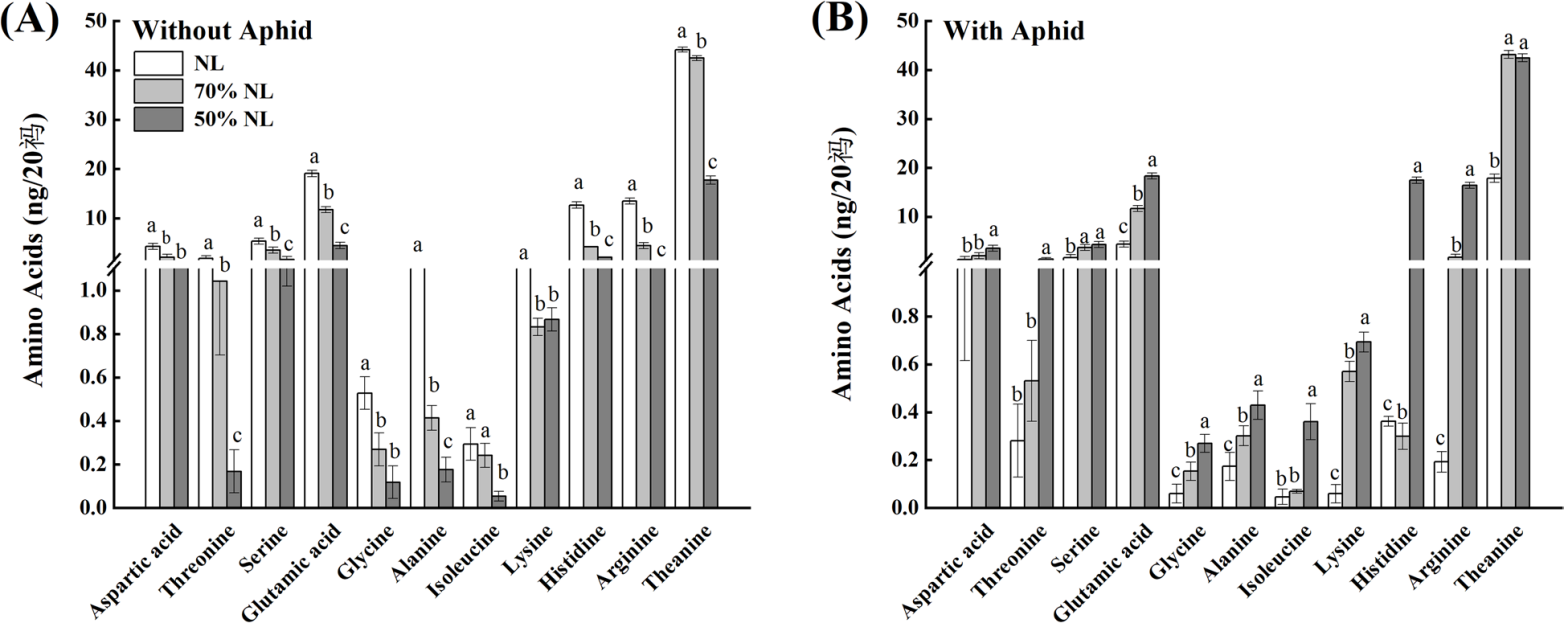
Foliar contents of the functional components; essential amino acids and theanine of tea seedlings without (**A**) and with (**B**) *T*. *aurantii* infestation for 30 days after 45 days grown under normal and reduced N-fertilizer levels

Compared with NL, 70%NL and 50%NL both significantly reduced the foliar contents of all the essential AAs except that of isoleucine (Isl) of tea seedlings without *T*. *aurantii* infestation (*P* < 0.05), and 50%NL significantly decreased the foliar content of Isl of tea seedlings without *T*. *aurantii* infestation (*P* < 0.05; Fig. 6A); Moreover, 70%NL and 50%NL both significantly enhanced the foliar contents of serine (Ser), glutamic acid (Glu), Gly, Ala, Lys and Arg of tea seedlings with *T*. *aurantii* infestation (*P* < 0.05), and 50%NL significantly increased the foliar contents of aspartic acid (Asp), threonine (Thr), Isl, and His of tea seedlings with *T*. *aurantii* infestation (*P* < 0.05), while 70%NL significantly decreased the foliar content of His of tea seedlings with *T*. *aurantii* infestation (*P* < 0.05; Fig. 6B).The foliar contents of Thr, Ser, Glu, Ala, Isl, His and Arg of tea seedling for the 50%NL were all significantly lower than those for the 70%NL under no aphid infestation (*P* < 0.05; Fig. 6A), and the foliar contents of all essential AAs except that of Ser of tea seedlings for the 50%NL were all significantly higher than those for the 70%NL under aphid infestation (*P* < 0.05; Fig. 6B).

#### Volatile organic compounds (VOCs)

**Fig. 7:**
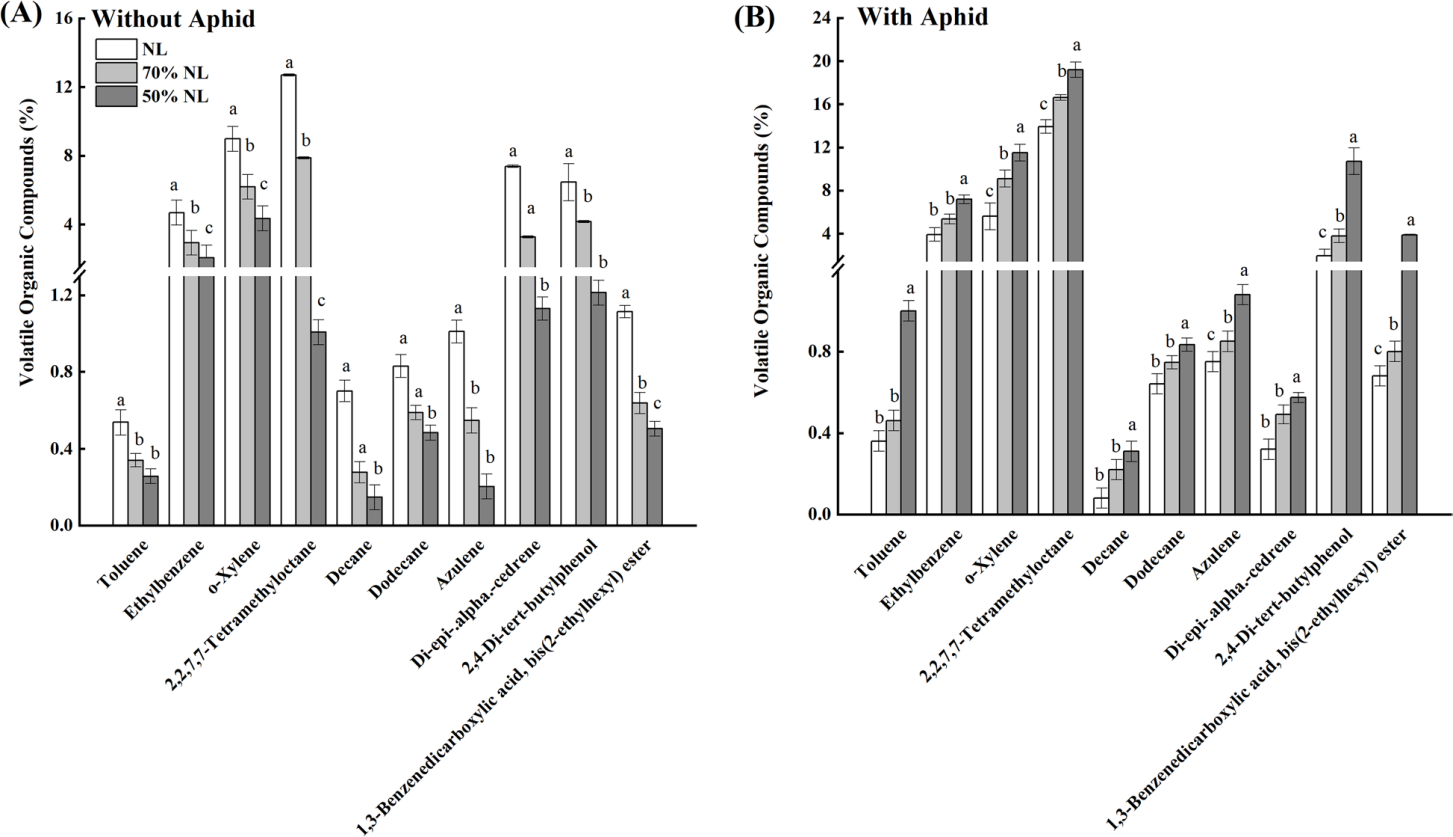
Percentage of volatile organic compounds (VOCs) in the leaves of tea seedlings without (**A**) and with (**B**) *T*. *aurantii* infestation for 30 days after 45 days grown under normal and reduced N-fertilizer levels (Note: TOL—Toluene; ETB—Ethylbenzene; OXL—0-xylene; TMO—2,2, 7,7- tetramethyloctane; DEC—Decane; DODEC—Dodecane; AZU—Azulene; DEACD—di-epi-alphacedrene; DTP—2,4-di-tert-butylphenol; BDCXA—1,3 benzenedicarboxylic acid; B2EHE—bis (2ethylhexyl) ester)

Total ten types of VOCs in the sampling leaves of tea seedlings were identified and their percentages are shown in Fig. 7. Aphid infestation and its interaction with N-fertilizer application both significantly affected the percentages of the measured ten types of VOCs in the leaves of tea seedlings (*P* < 0.001), and N-fertilizer application also significantly influenced the percentages of seven types of VOCs, including toluene (TOL), 2,2, 7,7- tetramethyloctane (TMO), decane (DEC), azulene (AZU), di-epi-alphacedrene (DEACD), 2,4-di-tert-butylphenol (DTP), and 1,3 benzenedicarboxylic acid (BDCXA)) in the leaves of tea seedlings (*P* < 0.001; Table 2).

Compared with NL, 70%NL and 50%NL both significantly reduced the percentages of toluene-TOL (-54% and -64%), ethylbenzene-ETB (-25.36% and -45.43%), 0-xylene-OXL (-20.92% and -51.32%), 2,2, 7,7- tetramethyloctane-TMO (-13.47% and -27.46%,), decane-DEC (-29.03% and -74.19%), dodecane-DODEC (-10.4% and -23.2%), azulene-AZU (-21.30% and -30.56%), di-epi-alphacedrene-DEACD,2,4-di-tert-butylphenol-2,4 DTP (-14.54% and -44.12%), 1,3 benzenedicarboxylic acid-1,3 BDCXA (-64.58% and -81.73%) and bis (2 ethylhexyl) ester-B2EHE (-79.43% and 82.52%) in the tea seedlings non-infested with tea aphid. In contrast, significant increases were found in the percentages of toluene-TOL (+ 24.51% and + 52.17%), ethylbenzene-ETB (+ 29.81% and + 56.09%), 0-xylene-OXL (+ 29.78% and + 51.61%,), 2,2, 7,7- tetramethyloctane-TMO (+ 87.21% and + 92.07%), decane-DEC (+ 46.99% and + 79.05%), dodecane-DODEC (+ 17.61% and + 41.77%), azulene-AZU (+ 62.80% and + 79.87%), di-epi-alphacedrene-DEACD,2,4-di-tert-butylphenol-2,4 DTP (+ 65.44% and + 84.70%), 1,3 benzenedicarboxylic acid-1,3 BDCXA (+ 70.81% and 81.21%) and bis (2ethylhexyl) ester-B2EHE (20.94% and 54.79%) in the tea seedlings infested with tea aphid in 70%NL and 50%NL compared to that in NL.

### Relative expression levels of synthetic genes of foliar functional components

The interaction of N-fertilizer application and aphid infestation remained significant, whereas N-fertilizer application didn’t significantly affect the relative expression level of *CsTCS, CsTS1* and *CsGT1* in tea leaves (*P* ≤ 0.05); (Table 2). The interaction between N-fertilizer application and aphid infestation significantly influenced the relative expression level of *CsTCs*, *CsTs1* and *CsGT1* in the leaves of tea seedlings (*P* = 0.001 < 0.05; Table 2).

**Fig. 8:**
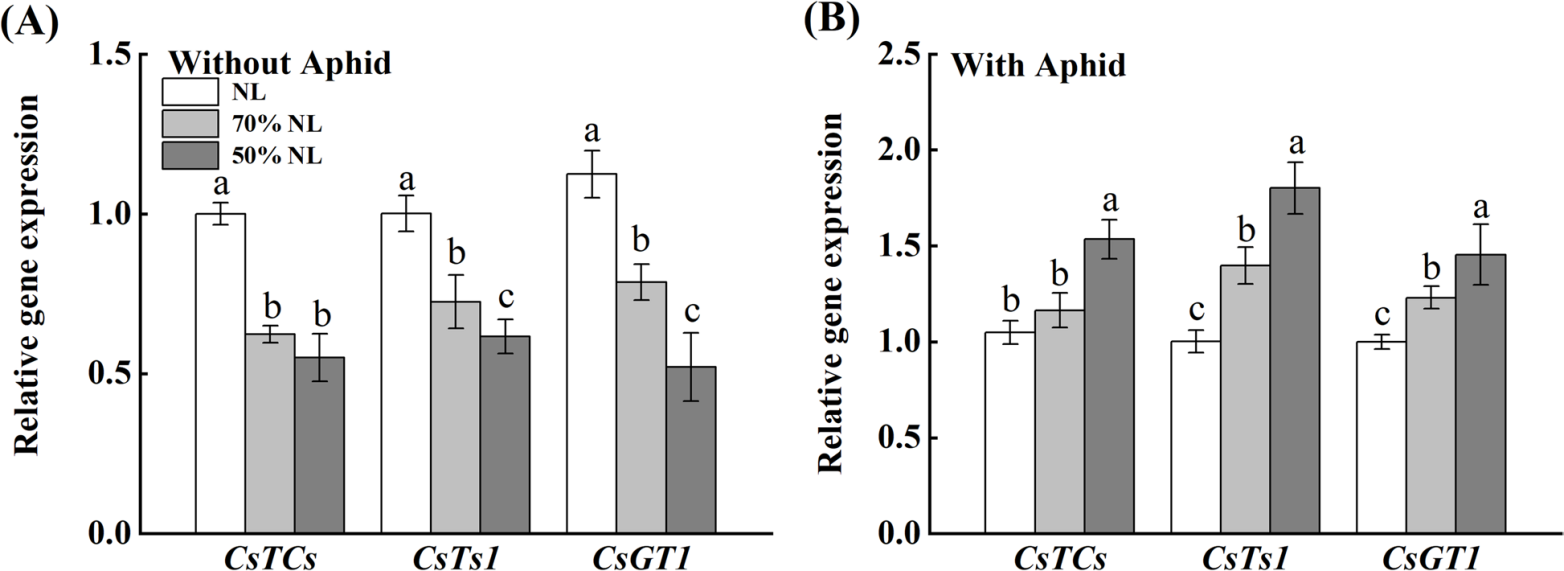
Relative transcript expression levels of the measured genes (including *CsTCs, CsTS1* and *CsGT1*) in the leaves of tea seedlings without (**A**) and with (**B**) *T*. *aurantii* infestation for 30 days after 45 days grown under normal and reduced N-fertilizer levels

Compared with NL, 70%NL decreased the transcript expression level of *CsTCs* (-0.10%), *CsTs1*(-16.21%) and *CsGT1*(-11.97%) in the tea seedlings non-infested with tea aphid. The 50%NL exhibited mixed responses where significant decrease in the relative transcript expression levels of *CsTCs* and *CsTs1* (-12.40% and -25.98%) was observed while significant increase in the relative transcript expression levels of *CsGT1* (+ 5.31%) was observed compared to NL. However, in the tea seedlings infested with tea aphid, 70%NL increased the relative transcript expression level of *CsTCs* (+ 4.51%) while significantly decreased that of *CsTs1* (-20.03%) and *CsGT1* (-17.39%) as compared to NL. Likewise, 50%NL increased the relative transcript expression level of *CsTCs* (+ 4.58%) and *CsGT1* (+ 5.25%) while significantly decreased the relative transcript expression level of *CsTs1* (-5.66%) in tea seedlings infested with tea aphid as compared to NL (Fig. 8).

### Population dynamics of tea aphids fed on tea seedlings grown under normal and reduced N-fertilizer levels

**Fig. 9:**
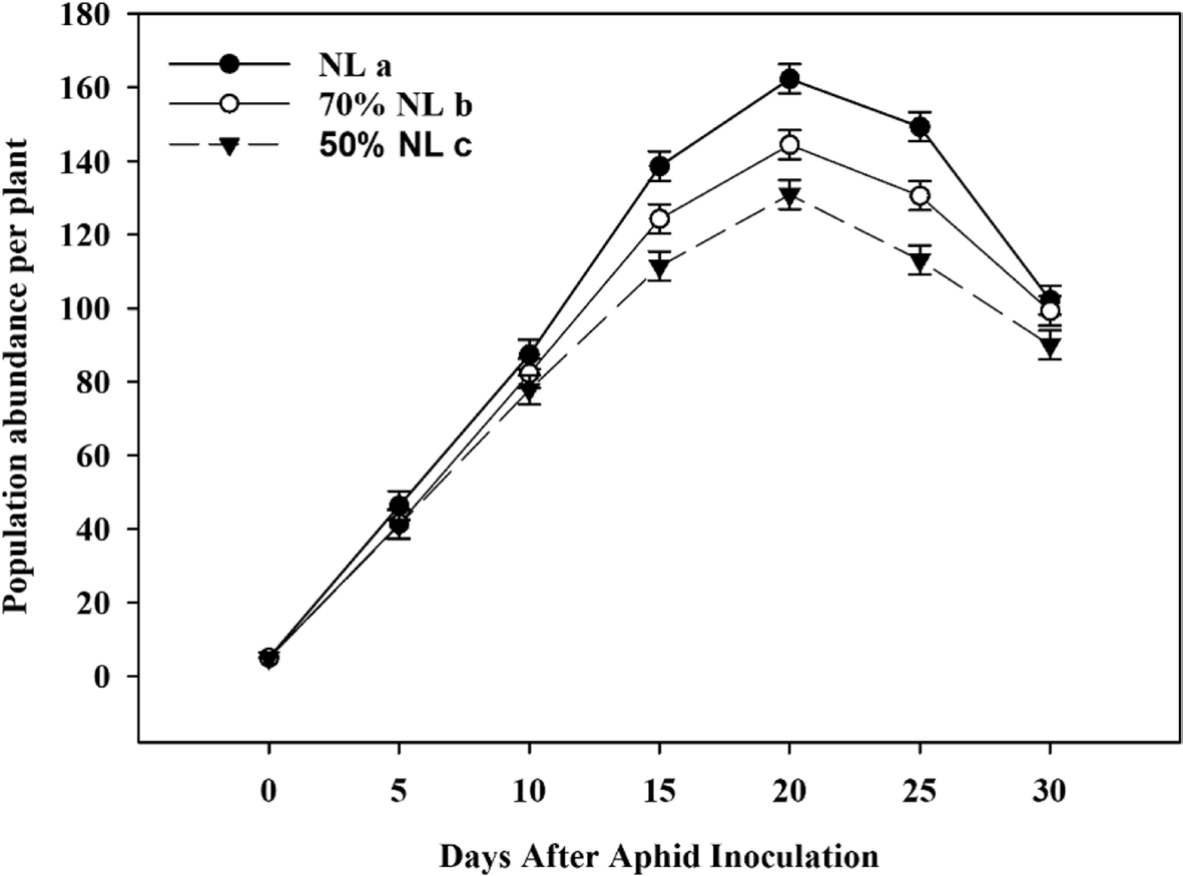
Population dynamics of *Toxoptera aurantii* fed on tea seedlings grown under normal and reduced N-fertilizer levels for 45 days (Note: One-way repeated-measures ANOVA with *F* = 177.4 and *P* < 0.001; Different lowercase letters indicated significantly different among three N-fertilizer levels of NL, 70%NL and 50%NL by LSD test at *P* < 0.05)

N-fertilizer application significantly affected the population dynamics of *T. aurantii* fed on tea seedlings (One-way repeated-measures ANOVA: *F* = 177.4, *P* < 0.001; Fig. 9). Compared with NL, 70%NL and 50%NL both significantly reduced the aphid abundance per plant on tea seedlings (*P* < 0.05), and there were significant difference in the aphid abundance per plant on tea seedlings between the two reduced-N fertilizer application treatments (*P* < 0.05; Fig. 9).

### Correlation analysis between plant growth, foliar soluble nutrients and functional components of tea seedlings and population abundance of *T. aurantii* under different N-fertilizer applications

**Fig. 10:**
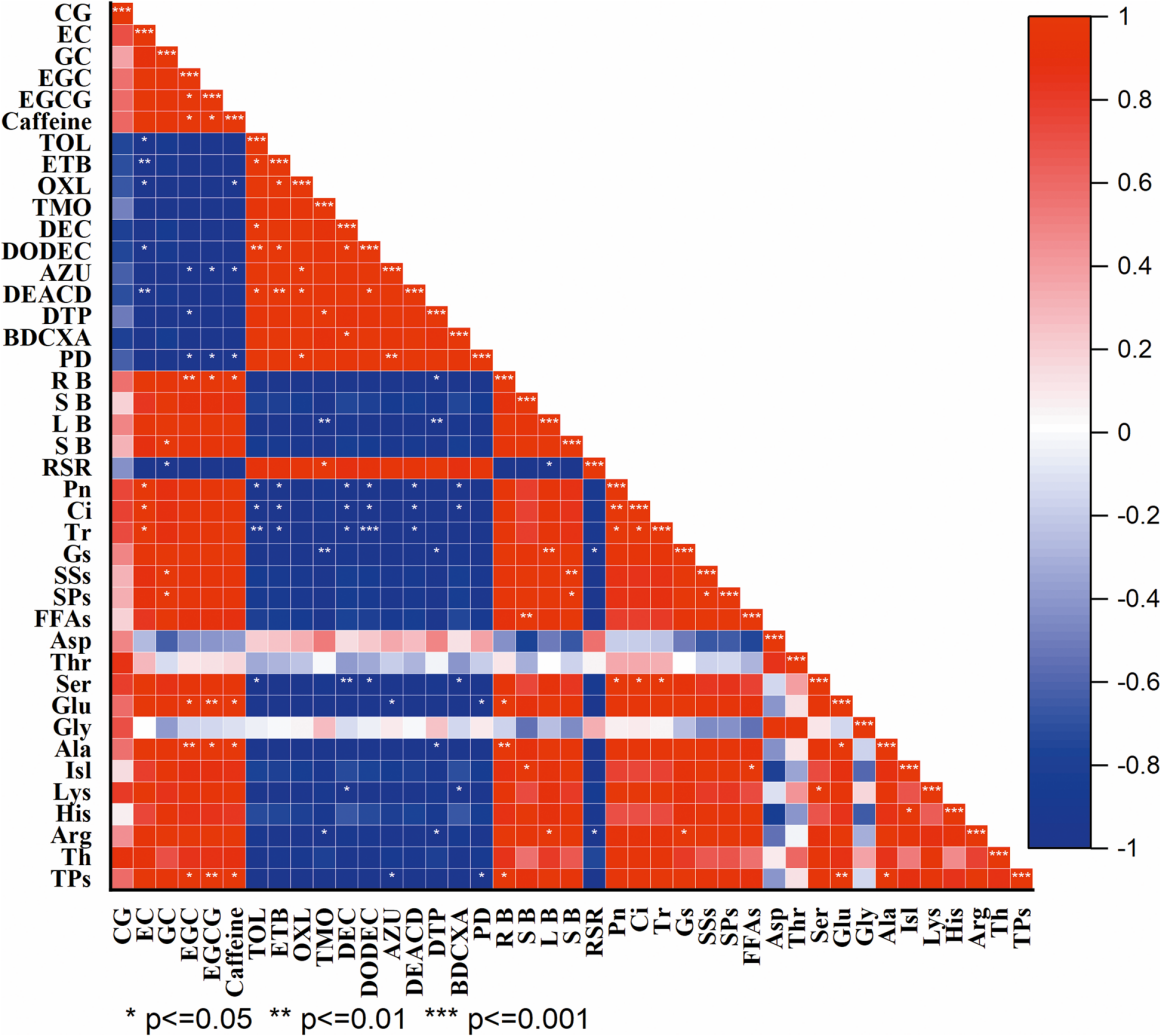
Correlation analysis of physiological parameters, foliar soluble nutrients and functional components of tea seedlings and population abundance of *T*. *aurantii* fed on tea seedlings grown under normal and reduced N-fertilizer levels (Note: Red color shade indicates positive correlation and blue indicates negative correlation. The darker the color, the stronger the correlation; PD—Population dynamics of *T. aurantii*; EC—Epicatechin; GC—Gallocatechin; EGC—Epigallocatechin; EGCG—Epigallocatechin gallate; CAFF—Caffeine; TOL—Toluene; ETB—Ethylbenzene); OXL—Oxylene; TMO—2,2, 7,7- Tetramethyloctane; DEC—Decane; DODEC—Dodecane; AZU—Azuelene; DEACD—Diepialphacedrene; DTP—2,4,-ditert-butylphenol; BDCXA—1,3 benzenedicarboxylic acid; RB—Root biomass; SB—Shoot biomass; RSR—Root shoot ratio; Pn—Net photosynthetic rate; Ci—intercellular carbon dioxide concentration; Tr—Transpiration rate; Gs—Rate of stomatal conductance; SSs—Soluble sugars; SPs—Soluble proteins; FFAs—Free fatty acids; Asp—Aspartic acid; Thr—Threonine; Ser—Serine; Glu—Glutamic acid; Gly—Glycine; Ala—Alanine); Isl—Isoleucine; Lys—Lysine; His—Histidine; Arg—Arginine; Th—Theanine; TPs—Total Polyphenols; CG—Catechin gallate. The same in the following figure)

The correlation analysis among all the studied parameters of all different treatments were performed by using new updated OriginPro 2021 (USA) (Fig. 10). The Pearson’s correlation values of all physiological and biochemical parameters of tea plants applied with various nitrogen doses (NL, 70%NL, and 50% NL) along with aphid inoculation showed that RSR, TOL, ETB, OXL, TMO, DEC, DODEC, AZU, DEACD, DTP, BDCXA were positively correlated with population dynamics of tea aphids (red color; Figs. 3-4 ), while TPs, Th, Arg, His, Lys, Isl, Ala, Glu, Ser, FFAs, SPs, SSs, Gs, Tr, Ci, Pn, SB, LB, SB, RB were negatively correlated with population dynamics of aphid (blue color; Fig. 10).

## Discussion

Nitrogen is the vital primary macronutrient that directly impacts the productivity and quality of tea plants and has been applied to improve the quality and yield of tea leaves [49, 50]. The quality constituents of green tea are comprised of amino acids, phenolic compounds, and caffeine and the quality rely on the abundance of these compounds in the young shoots [8, 51]. In this study, we evaluated the effects of two reduced N-fertilizer levels (i.e., 70%NL and 50%NL) with normal N-fertilizer level (i.e., NL; 240 kg N ha^−1^) as the control to quantify the effects of reduced N-fertilizer application on the physiological growth parameters including photosynthetic indexes, foliar soluble nutrients and foliar functional components, including relative transcript levels of foliar synthetic genes, and emission of VOCs with and without aphid inoculation. Additionally, population abundance of *T. aurantii* feeding on different nitrogen treatments was also examined.

### Biomass

Increased N supply is believed to be the trigger for an augmented biomass production of young shoots of plants. Bonifas and Lindquist [52] reported that there is always an interaction between aboveground and belowground competition for the resources and N supply. Cheruiyot et al.[53] mentioned that fertilizer supply remarkably influenced dry matter partitioning as per the theory of plant allocation strategies mentioned by Ågren [54]. Ågren and Ingestad [55] further mentioned that the root/shoot ratio (i.e., RSR) increases with increased N level. Dry matter portioning or the RSR is defined as dry weight of root biomass divided by dry weight of shoot biomass and it depends upon the partitioning of photosynthates which might be altered by environmental stimuli [56]. According to Bhattacharya [57], unavailability of phosphorus (P), one of the essential macronutrients, normally leads to higher RSR and overall changes in root framework. In our experiment, NL showed increased aboveground (shoot) and belowground (root) biomass of tea seedlings which is consistent with the study by Cheruiyot et al. [53] as compared to reduced N-fertilizer levels. Moreover, the RSR of NL was larger than that of 70% NL and 50% NL. However, the trend on increment of biomass for the aphid inoculated tea followed the reverse order, reduced N-fertilizer dose, specifically 50% NL, showed largest increment in biomass as compared with NL and 70%NL. Also, the RSR of 50%NL was larger than that of NL and 50% NL in the aphid inoculated tea seedlings.

### Photosynthetic indexes

The rate of photosynthesis is impacted by nitrogen nutrition in plants. Here, four photosynthetic indexes have been measured including Pn, Gs, Ci and Tr. Nitrogen treated tea seedlings for 45 days showed significant increase in all the four aforementioned photosynthetic measures which is consistent with the study by Osman [58], who reported that the increase in the gross photosynthesis was linear with nitrogen content. Normal N showed significant effects on Pn, Gs, Ci and Tr as compared to reduced N treatments. Augmented N content enhanced chlorophyll contents in black tea which would eventually increase Pn rate due to the stimulation of photosynthetic pigments [59, 60]. According to Cheng et al. [61], N abundance can regulate plant photosynthetic activity and the formation of products due to the fact that more carbon present in the leaves would be assimilated into amino acids and proteins in lieu of carbohydrate. On the other hand, in the 30 days’ aphid inoculated tea seedlings, reduced N treatment (50%NL) showed significant increases in all four parameters as compared to those of NL and 70%NL since the aphid abundance was less in 50%NL as compared to that in NL and 70%NL.

### Foliar soluble nutrients (SSs, SPs, FAAs)

Alterations in the relative content of chemical constituents are the most predominant scene in tea plants after the synthetic N incorporations [62]. Moreover, Owuor et al. [63] mentioned the significance of N in increasing the levels of fatty acids. Tea seedlings with three N-fertilizer treatments for 45 days showed changes in the foliar nutrients; SSs, Sps and FFAs where NL showed significant changes with the increase in concentration for all the above mentioned foliar soluble nutrients (SSs, SPs & FFAs) as compared to 70%NL and 50%NL which is consistent with the results of [62, 63]. Soluble sugar concentration was increased and then decreased steadily with the increment of synthetic N as reported by Qiao et al. [62]. However, N treated tea seedlings inoculated with aphids for 30 days showed the alterations being noted on a reverse trend where reduced N treatment significantly increased foliar contents as compared to normal and 70%NL.

### Foliar functional components

Polyphenols are the major phytochemicals in tea, and plays decisive role in the color, aroma and taste of tea [64]. Flavonoids are the most important phenolic compounds which impart color, taste and aroma and the pre-eminent flavonoids are catechins; (-)-epigallocatechin gallate (EGCG), (-)- epigallocatechin (EGC), (-)-epicatechin gallate (ECG) and (-)-epicatechin (EC) [65, 67]. In our study, the 14-month-old tea seedlings were treated with three N treatments, where NL significantly enhanced the polyphenol concentration as well as the percentage concentration of GC, EGC, EGECG, EC, CG as compared to reduced fertilizer rates (70%NL and 50%NL). Our findings were consistent with the studies by Venkatesan et al. [12] who reported significant increase of TPs, EGC, EGCG and EC contents with the increase of nitrogen [66]. Moreover**,** in bush tea *(Athrixia phylicoides)*, the chemical concentration of total polyphenols, total flavonoids and total antioxidants of bush tea leaves clearly showed increment with response to nitrogen nutrition. Furthermore, the tea seedlings inoculated with aphid infestation for 30 days, 50%NL showed the enhanced concentration of polyphenols as well as tea catechins including GC, EGC. EGECG, EC, CG as compared to those in normal and 70%NL.

In green tea, AAs contribute to the mellowness and freshness infusion where Glu and Thea are of considerable significance [9]. Several studies reported that incorporation of N elevates concentrations of free amino acids but no clear indication of how Thea is affected by external N supply [7]. In our study, we measured the concentration (ng/20 µl) of ten essential amino acids and theanine. And for all the essential amino acids (including theanine), NL showed the enhanced concentrations as compared to 70%NL and 50%NL which was consistent with the prior study by Ruan [7]. However, it showed the reverse trend for the tea seedlings inoculated with tea aphids due to the preference of aphid to enhanced soluble nutrient contents of essential amino acids and theanine as the choice displayed NL > 70%NL > 50%NL, where reduced N-fertilizer treatment (i.e., 50%NL) was the least affected and hence the concentration was also significantly enhanced. On organismal level, reduced level of nitrogen application (lower than optimum rates) negatively impacts both vegetative growth and reproductive performance of the host plant, thereby negatively impacting the aphid population dynamics. These effects clearly manifest at the plant physiological level as shown in our present study.

Caffeine, an alkaloid containing N is one of the vital functional quality components that can affect the tea quality characteristics confabulating astringency and bitterness [68–70]. Hamid et al. [73] reported that different N treatments did not significantly affect the quality of caffeine, an important green tea quality determinant. Correspondingly, less caffeine production was reported with less N incorporation during the interval of 14 months in tea plants; however, the elevated N application imparted superior caffeine contents [71, 72]. Likewise, in our study of three N treated 14-month tea seedlings kept for 45 days, caffeine showed significant responses to different N treatments. Though the concentration of caffeine did not differ across treatments, NL showed significantly higher caffeine concentration as compared to reduced N treatments which is similar to the findings by [71, 72]. However, the tea seedlings inoculated with tea aphids in NL attracted more aphids and hence the concentration showed the reverse trend where the concentration of caffeine was significantly higher in 50%NL compared with NL and 70%NL.

### Foliar functional genes

There are several foliar functional genes involved in the biosynthetic pathways of theanine and caffeine, genes responsible for floral aroma, genes responsible for anthocyanin regulatory mechanism, and the genes responsible for the emission of tea volatiles. We took the four essential key synthetic genes and studied their relative transcript expression levels. The genes thus studied were *CsTCs* (genes responsible for caffeine), *CsTs1* (genes responsible for theanine) and *CsGT1* (genes responsible for tea VOCs emission). In the three different N-treated 14-month-old tea seedlings studied for 45 days, reduced N treatment (70%NL) expressed significantly higher relative transcript expression level of *CsTCs, CsTs1* and *CsGT1*, compared to NL and 50%NL. Moreover, for the tea seedlings with aphid inoculated for 30 days, 70%NL showed higher transcript expression level of *CsTCs* and *CsTs1* compared to NL and 50%NL.

### VOCs

Volatile compounds (VOCs) are influential defensive compounds which are released by plants against insect herbivore attack and affected by several biotic and abiotic stresses [74–76]. During the recent years, study of tea VOCs or aromatic components have also gained momentum as they are also one of the pivotal constituents in determining the quality of tea [77]. According to Odak et al. [78], the increment of N-fertilizer application increases green leaf volatiles (GLVs) while decreased aromatic and terpenoid compounds. They further mentioned that ethyl benzene and ß-cedrene were decreased with the N-fertilizer applications. Studies reported that when combined with N hazardous pollutants like toluene and benzene indirectly influence climate changes and benzene compounds are used as insect repellents [79–81]. Also, azulene is an aromatic hydrocarbon which is well known for its role as a plant metabolite, insect repellent, and phytotoxic properties [82, 83]. Di-epi-α-cedrene belongs to the class of terpene and derivatives aromatic compounds, and terpene is a phyto-compound [84]. Terpenes are essential building blocks in plant biochemistry and include those groups of plant secondary metabolites which attract pollinators and protect plant from foreign matter invasion including microorganisms [85, 86]. The 2,4-Di-tert-butylphenol (2,4-DTBP) is a prevalent toxic secondary metabolite produced by various groups of organisms which shows remarkable adulticidal, larvicidal, ovicidal, repellent, and oviposition-deterrent actions against the spider mite, *Tetranychus cinnabarinusn* [87, 88]. Moreover, 2,4-DTBP, a toxic lipophilic phenol which has been identified in bacteria, algae, fungi and plants, and insects also exhibited nematicidal activity against *Caenorhabditis elegans* during fumigation or soil treatment at temperatures higher than 25 °C [87, 89]. Similarly, 1,3 benzene dicarboxylic acid belongs to the class of phthalic acid esters (phthalates) and are the simplest aromatic dicarboxylic acids and found in beverage/food samples, such as fruit juices and tea-based drinks [90, 91]. While bis (2 ethylhexyl) ester belongs to the class of phthalate esters (PAEs) and its occurrence is similar to phthalates. Dodecane and decane belongs to the class of alkane hydrocarbons. Dodecane, a liquid alkane hydrocarbon, has been reported as an aromatic volatile in green tea and Oolong tea and also a little proportion is reported in Pu-erh ripe tea [92, 93]. Decane is also an alkane hydrocarbon, observed in Turkish green tea powder and is generally believed to be fairly nontoxic, compared to other aliphatic hydrocarbons [94]. Decane also holds a significant position in insect bio-control where it is used as an alarm pheromone of the ant *Camponotus obscuripes* [95].

The ten major VOCs thus detected in our study were grouped into aromatic hydrocarbons, terpenes and its derivatives, alkane hydrocarbons, phthalic acid esters (phthalates) and phthalate esters (PAEs). The mono-aromatic hydrocarbons, abbreviated BTEX consists of benzene, toluene, ortho, meta and para xylene isomers and styrene [96]. Compounds toluene, ethylbenzene, 0-xylene, 2,2, 7,7-tetramethyl-octane and azulene belong to the class of an aromatic hydrocarbons while toluene, ethylbenzene and 0-xylene belong to the class of mono aromatic hydrocarbons. Ten common VOCs prevalent in tea were identified and their percentage compositions were calculated; Normal N level significantly affected the emission of VOCs as compared to reduced fertilizer levels. NL significantly affected toluene, ethylbenzene, 0-xylene, 2,2, 7,7-tetramethyloctane, decane, dodecane, azulene, di-epi-alpha cedrene, 2,4-di-tert-butylphenol,1,3 benzenedicarboxylic acid and bis (2ethylhexyl) ester. Also, reduced N-fertilizer treatments exhibited significantly lower percentage concentrations of VOCs emission. Likewise, NL attracted more tea aphids, and the emission of VOCs was more in NL compared to that in 70%NL and 50%NL. For the aphid inoculation treatments, NL significantly affected the emission of VOCs, including toluene, ethylbenzene, 0-xylene, 2,2, 7,7-tetramethyloctane, decane, dodecane, azulene, di-epi-alpha cedrene, 2,4-di-tert-butylphenol,1,3 benzenedicarboxylic acid and bis (2ethylhexyl) ester, compared to that in 70%NL and 50%NL, and 50%NL showed the significant emission of VOCs as compared to NL and 70%NL.

## Summary

**Fig. 11:**
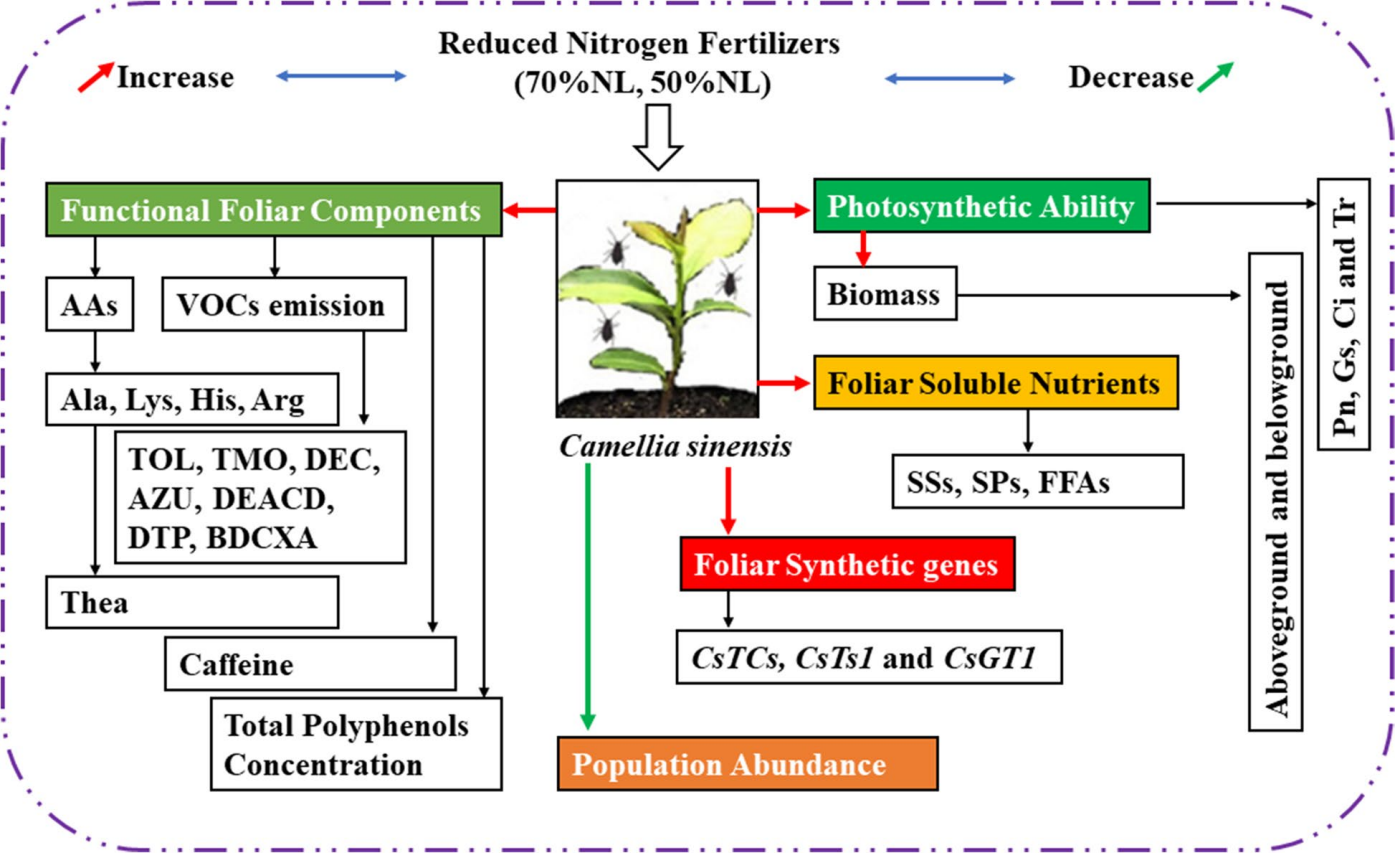
Schematic diagram of reduced nitrogen treatments and its effects on tea quality indexes, growth and development, and population dynamics of tea aphids

Normal N-fertilizer level (i.e., NL) enhanced the biomass, RSR, photosynthetic indexes, foliar soluble nutrients (SSs, SPs, FFAs, FAAs), foliar functional components (caffeine, AAs, tea catechins, concentration of TPs and VOCs emission) in the 14-month-old tea seedlings without tea aphid inoculation compared to 70%NL and 50%NL. Also, 50% NL showed significant results in the enhancement of the above measured indexes of tea seedlings with tea aphid inoculation as compared to NL and 70%NL. The deterioration of the quality components in the NL and 70%NL is due to the attraction of tea aphids to the lusher plants that received more N than other treatments and hence the tea aphid attack was more and also the deterioration in the physiological parameters, foliar soluble components, foliar functional components and VOCs. On volatile emission, volatile concentration on NL was higher in the aphid inoculated tea seedling since it was mostly damaged by aphids and also tea seedlings went under stress after the aphid feeding. Reduced N-fertilizer applications (70%NL and 50%NL) significantly decreased the percentage concentration of VOCs in the tea seedlings without aphid infestation, while concomitantly increased the percentage concentration of VOCs in tea seedlings with aphid infestation. Moreover, the population abundance of tea aphids in NL treatment was higher than those in 70%NL and 50%NL. The results clearly demonstrated that the reduced N-fertilizer applications could enhance VOCs of tea plants especially with *T. aurantii* infestation and the pest population dynamics also diminished in reduced nitrogen fertilizer treatments which may, in turn, require lower pesticide use for control of tea aphid infestation in tea plantations. Also, practice of reducing N-fertilizer application serves in the interest of ecological balance and sustainability. The schematic model has been proposed based upon the researches hitherto upon the tea seedlings, based on consideration of N fertilizer, metabolomics, essential foliar functional and foliar components, foliar functional genes and functional volatiles and its interaction with aphid (seen in Fig. 11).

N-fertilizer has positive role in tea (*Camellia sinensis* L*.*) with the enhancement of tea biochemical contents and tea physiological indexes but the attraction of notorious tea aphids to higher N-fertilizer application is also of prime concern since tea aphids damage the quality of tea by altering the concentration of foliar soluble and foliar functional components. So, a further research is warranted on the tea aphids and N fertilizers; as in aphid inoculated treatments reduced N-fertilizer applications enhanced the tea quality parameters, while in tea seedlings without tea aphid inoculation, the normal N-fertilizer application showed remarkable effects. Also, since the transcript expression levels of foliar functional genes was elevated in the reduced N-fertilizer treatments in both with and without tea aphid infested plants, further research is warranted in the tea foliar functional genes and synthetic N. As the threat of climate change is looming and tea aphid infestation will also be on the rise along with the emergence of new pests, maintaining sustainability in the ecosystem in accordance with the lower use of synthetic N fertilizer is a big challenge for an already booming tea industry.

## Data Availability

The datasets generated and/or analyzed during the current study are available from the corresponding author upon reasonable request.

## Abbreviations

N: Nitrogen
C: Carbon
P: Phosphorus
CO_2_: Carbon dioxide
kgNha^−^^1^: Kilogram nitrogen per hectare
eV: Electron volt
ng: Nanogram
mm: Millimeter
NIST: The national institute of standards and technology
NL: Normal nitrogen level
RSR: Root shoot ratio
VOCS: Volatile organic compounds
Pn: Net photosynthetic rate
Gs: Rate of stomatal conductance
Ci: Intercellular CO_2_ concentration
Tr: Transpiration rate
μm: Micrometer
DNA: Deoxyribose-nucleic acid
RNA: Ribose nucleic acid
RH: Relative Humidity
min: Minute
rpm: Revolutions per minute
hr: Hour
cm: Centimeter
AAs: Amino acids
Asp: Aspartic acid
Arg: Arginine
Thr: Threonine
Ser: Serine
Ala: Alanine
Glu: Glutamic acid
Gly: Glycine
Ile: Isoleucine
His: Histidine
Lys: Lysine
Thea: Theanine
EGC: Epigallocatechin
EC: Epicatechin
EGCG: Epigallocatechin gallate
ECG: Epicatechin gallate
RT-PCR: Reverse transcriptase polymerase chain reaction
HPLC: High performance liquid chromatography
µl: Microliter
µg: Microgram
mg: Milligram
mL: Milliliter
cDNA: Complementary DNA
qRT-PCR: Quantitative reverse transcription polymerase chain reaction
µM: Micrometer
h: Hour
*CsTCs*: Caffeine synthase gene
*CsTs1*: Theanine synthase gene
*CsGT1*: Glycosyltransferase 1
TOL: Toluene
ETB: Ethylbenzene
OXL: 0-Xylene
TMO: 2,2, 7,7- Tetramethyloctane
DEC: Decane
DODEC: Dodecane
AZU: Azulene
DEACD: Di-epi-alphacedrene
DTP: 2,4-Di-tert-butylphenol
BDCXA: 1,3 Benzenedicarboxylic acid
B2EHE: Bis (2ethylhexyl) ester
